# Case Report: Endobronchial Foreign Body Retrieved via Flexible Bronchoscopy 51 Years After Initial Aspiration

**DOI:** 10.1155/crpu/9666495

**Published:** 2026-08-03

**Authors:** Saif-El-Din El-Akkad, Michael Brown, Renelle Myers

**Affiliations:** ^1^ Department of Medicine, Division of Respirology, University of British Columbia, Vancouver, Canada, ubc.ca; ^2^ Department of Integrative Oncology, BC Cancer, Vancouver, British Columbia, Canada, bccancer.bc.ca

**Keywords:** airway foreign body, aspiration, bronchoscopy, retained foreign body

## Abstract

Undiagnosed and retained aspirated foreign bodies are a well‐recognized cause of pulmonary morbidity, yet diagnosis of this clinical entity is often delayed, particularly when the foreign body is radiolucent, or the history of aspiration cannot be recalled. We report the longest documented case of a retained airway foreign body to date: a 59‐year‐old woman who aspirated a plastic Barbie doll house cup at age 8, 51 years prior. She experienced recurrent right lower lobe (RLL) pneumonias, and CT imaging eventually revealed chronic RLL collapse and bronchial obstruction. Flexible bronchoscopy confirmed and enabled successful removal of the foreign body. This case underscores the importance of maintaining clinical suspicion for foreign body aspiration in adults with persistent or recurrent respiratory symptoms, even when the initial aspiration event occurred many years prior. It also highlights the diagnostic challenges associated with radiolucent foreign bodies and the potential for long‐term pulmonary complications when diagnosis is delayed.

## 1. Introduction

Foreign body (FB) aspiration is a well‐established condition in children and, less commonly, adults [1–3]. Peak incidence occurs in children aged 2–3 years old and adults over 60 [2, 4]. FBs lodged within bronchi may be minimally symptomatic or associated with a cough, unilateral wheeze, and recurrent pneumonias. Misdiagnosis as chronic respiratory disease is common and results in diagnostic delays and morbidity [3, 5]. Early retrieval of the FB via bronchoscopy is crucial to alleviating symptoms and preventing complications [1, 5].

Although rare, cases of retained airway FBs for 40–45 years have been documented [1, 3, 6]. We present an unusual case of a 59‐year‐old woman with an aspirated FB retained for 51 years, the longest documented case in the medical literature. This case highlights the importance of careful clinical assessment and the impact of diagnostic biases.

## 2. Case Report

A 59‐year‐old woman was referred to respirology to rule out an endobronchial lesion in the right lower lobe (RLL). She reported an 8 pack‐year smoking history and had stopped smoking 31 years prior to our initial evaluation. Her history was significant for recurrent pneumonias in childhood requiring multiple courses of antibiotics over the span of a few years. These respiratory symptoms eventually subsided in early adolescence, and she remained reasonably well thereafter for multiple decades.

**Figure 1:**
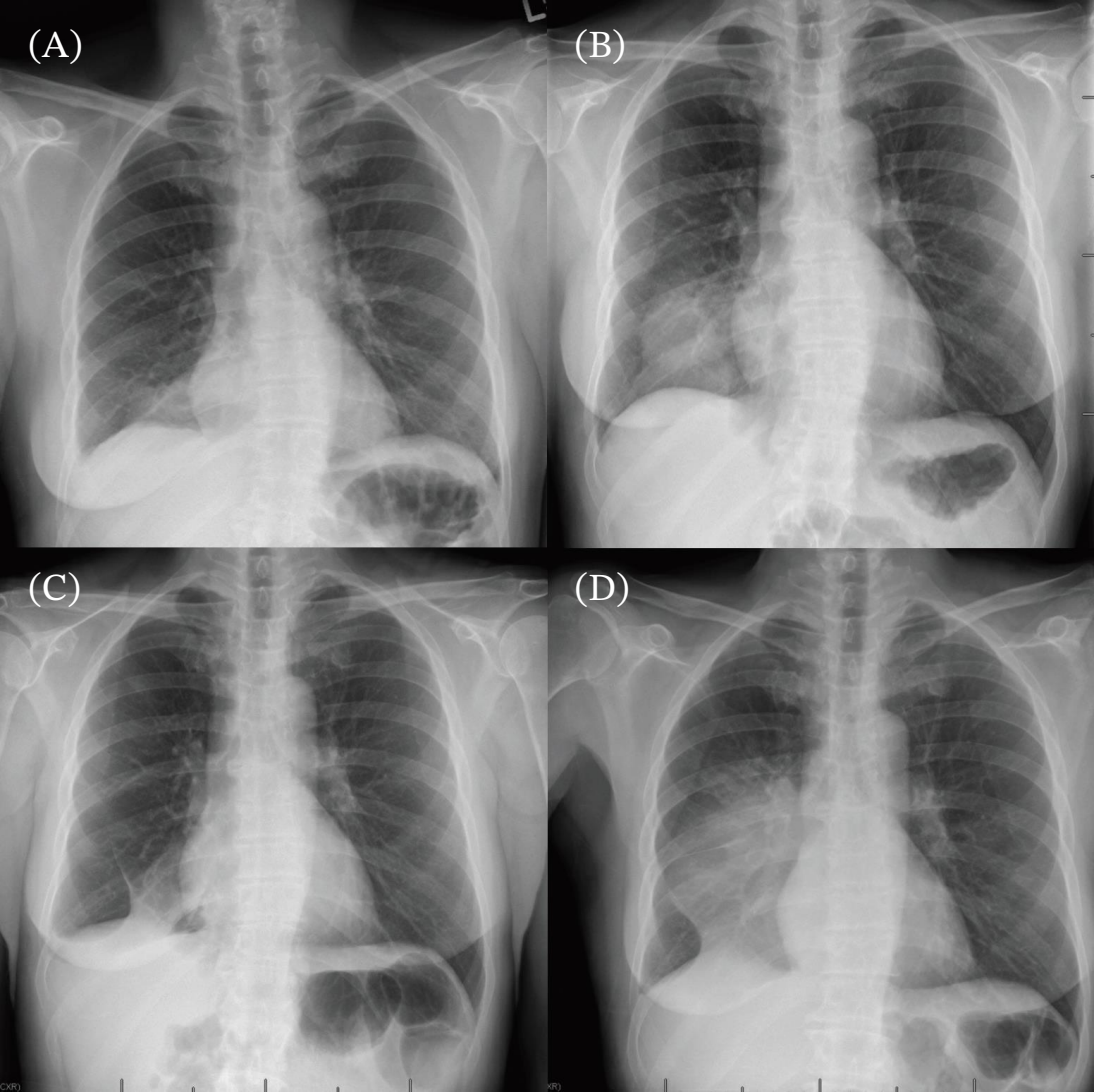
Chest X‐ray series spanning (A) 8 years prebronchoscopy, (B) 5 years prebronchoscopy, (C) 18 months prebronchoscopy, and (D) 12 months prebronchoscopy.

**Figure 2:**
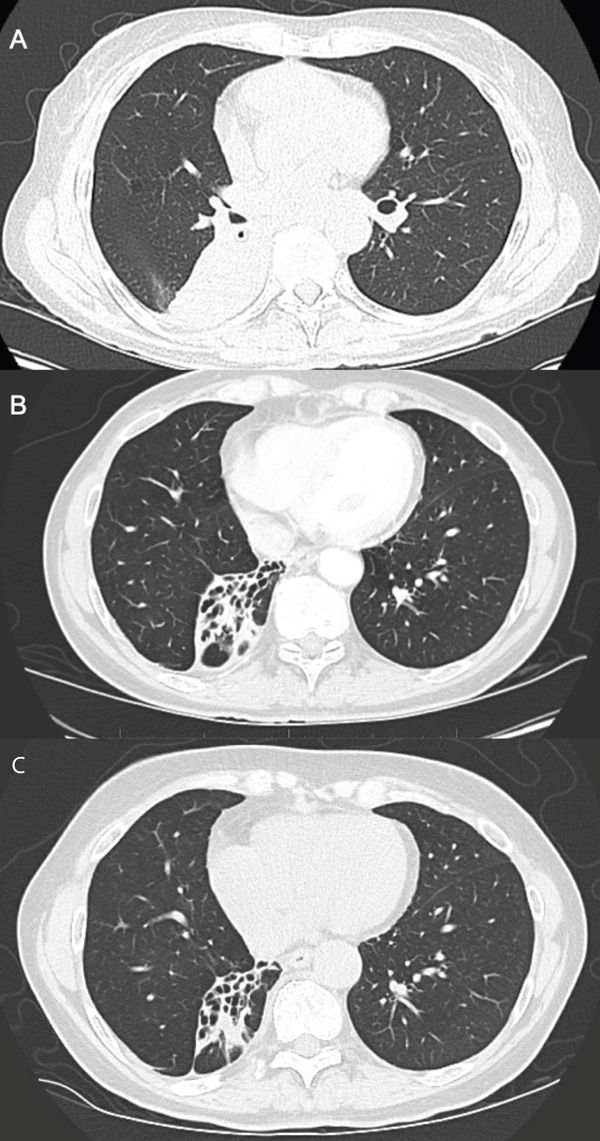
(A) CT chest 2 weeks prebronchoscopy, (B) CT chest 2 months postbronchoscopy, and (C) CT chest 9 months postbronchoscopy.

In the years prior to her referral, frequent and severe episodes of RLL pneumonia re‐emerged. These pneumonias resulted in numerous hospital visits with chest radiographs spanning an 8‐year period demonstrating waxing and waning RLL consolidation/atelectasis (Figure 1). These presentations were attributed to her prior smoking history and a potential diagnosis of chronic obstructive pulmonary disease (COPD) which resulted in significant diagnostic delay and bias. The patient ultimately received five courses of antibiotic therapy in the 8 months prior to our evaluation and given the increasing frequency and severity of her symptoms, and a CT chest was eventually obtained. CT chest revealed RLL consolidation, volume loss, and obliteration of the lateral (RB9) and posterior (RB10) basal bronchi (Figure 2), and the patient was referred to respirology for further evaluation of potential lung cancer.

Upon our evaluation, she recalled aspirating a small plastic cup from a Barbie dining set at age 8 which precipitated the aforementioned respiratory issues in childhood. She had reported this to physicians as a child and again repeatedly over the years as an adult, but radiographs failed to detect the radiolucent object, and she was incorrectly led to believe that it had been expectorated.

**Figure 3:**
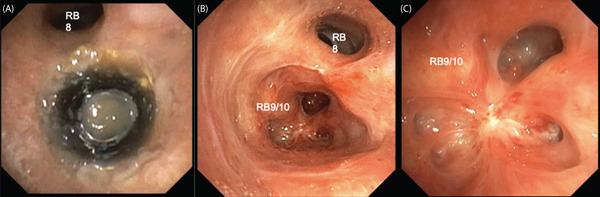
(A) Bronchoscopy image of retained foreign body. (B) Stenotic airways 2 months post foreign body retrieval, RB 8, 9, and 10 are shown. (C) Stenotic airways 2 months post foreign body retrieval, RB 9 and 10 are shown.

**Figure 4:**
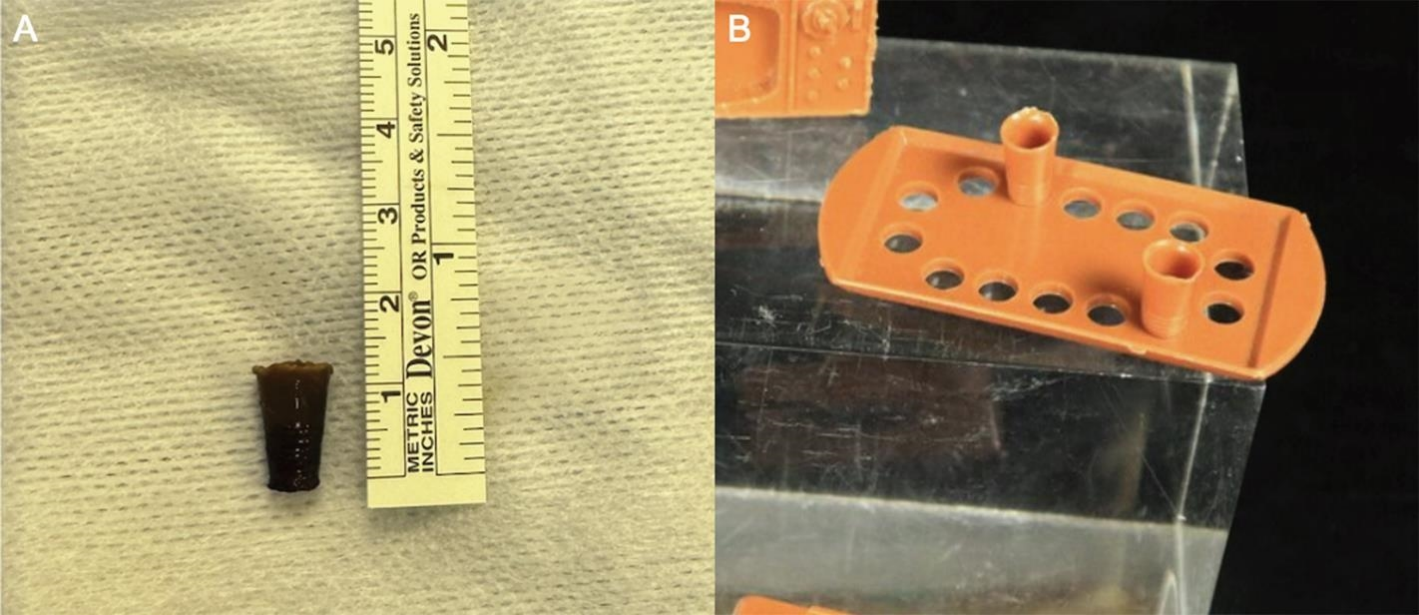
(A) 51‐year‐old retrieved foreign body. (B) 1970’s Mattel Barbie cup with identical lower and upper ridges and shape.

Bronchoscopy subsequently revealed an FB completely obstructing RB9 and RB10 consistent with her history and prior imaging (Figure 3). It was successfully removed using forceps and a basket (Figure 4) [7]. Biopsies of the tissue surrounding the FB were consistent with granulation tissue and not malignancy.

Follow‐up bronchoscopy 6 weeks post removal revealed improved granulation tissue but a severely stenosed RB9 and RB10. A CT chest repeated 2 months post removal demonstrated improved consolidation, residual bronchiectasis, and RLL volume loss. Subsequent CT chest 9 months post removal demonstrated stable albeit persistent RLL bronchiectasis, scarring, and volume loss. Despite persistent pathology on imaging, on follow‐up evaluation, the patient did not endorse any persistent respiratory complaints or limitation, and therefore, further surgical intervention (e.g., airway dilatation) was deferred. Re‐referral to respirology for surgical intervention was recommended should the patient develop recurrent RLL pneumonia.

## 3. Discussion

While rare, some airway FBs have remained undiagnosed for many decades [1, 3, 6]. To our knowledge, this is the longest documented case of retained airway FB at over 50 years.

Diagnosis of an aspirated FB requires a high index of suspicion. Symptoms can be nonspecific: most commonly cough (59%–94%), wheezing (22%), dyspnea (13%–41%), fever (18%–23%), hemoptysis (10%–23%), and pneumonias [1, 2, 5]. Surprisingly, many patients may not recall the aspiration event. Rates of recall range from 50% to 90% and are as low as 30% in the elderly [5, 8, 9]. In adults, symptoms may resolve spontaneously following aspiration, or only mild and underreported residual symptoms may persist. Physical exam findings, if present, may include stridor, intermittent unilateral wheezing, hyperinflation, or volume loss [1, 2, 5]. A minority of cases may also present with pneumothorax due to a check‐valve mechanism in the context of bronchial obstruction; the tapering nature of the FB in this case may have helped mitigate this complication by securely fitting in the airway [5]. Long‐term complications of retained foreign bodies include recurrent pneumonias, lung abscesses, bronchiectasis, and bronchostenosis or stricture formation due to the development of granulation tissue as demonstrated in Figures 1, 2, and 3 [5].

Overreliance on imaging alone may also lead to misdiagnosis. It is estimated that only 21%–32% of FBs are visible on chest radiograph [3, 5]. Many FBs are radiolucent, and the diagnosis relies on indirect radiographic findings like atelectasis or consolidation, particularly of the RLL in adults [2, 5, 8]. In adults, aspirated FBs tend to lodge in the right‐sided tracheobronchial tree in 60%–70% of cases; this is due to the anatomy and orientation of the right main bronchus—which is steeper and wider than the left main bronchus—at the level of the carina [8, 10]. Once an FB is suspected, then a CT chest should be obtained to aid with diagnosis and procedural planning [1, 3, 5]. Children or adults suspected of an FB aspiration or presenting with unexplained recurrent pneumonias should be proactively considered for bronchoscopy.

Our case unfortunately also highlights gender‐based diagnostic biases in women′s healthcare. Despite clearly reporting the aspiration event, her symptoms were dismissed, and she was never referred for definitive workup. This delay led to preventable long‐term pulmonary complications. Studies show that women are more likely to be misdiagnosed or face diagnostic delays compared to men in a variety of acute and chronic respiratory and nonrespiratory disease processes [11–13].

In our case, despite presenting with a classic history, including a reported aspiration event and recurrent RLL pneumonias, the patient was misdiagnosed and treated for COPD and community‐acquired pneumonias instead. Ultimately, multiple clinical pitfalls highlighted throughout this case report likely contributed to a delayed diagnosis in this patient, including (1) their prior smoking history and early anchoring to a diagnosis of COPD, (2) the presence of a radiolucent FB, (3) a remote aspiration event in childhood with delayed complications worsening primarily in adulthood, and (4) gender‐based diagnostic biases.

In conclusion, this case highlights the importance of careful history taking and physical examination in patients with persistent respiratory symptoms. Airway FBs are easily missed, leading to preventable complications. Various clinical pitfalls and diagnostic biases occurring over the span of decades resulted in this aspirated FB being retained for 51 years, the longest documented case in the medical literature. Bronchoscopy for definitive diagnosis should be considered early when the history or imaging is suggestive of aspiration. This case also highlights the impact of diagnostic bias and the importance of equitable care for patients of all genders.

## Funding

No funding was received for this manuscript.

## Ethics Statement

Written informed consent was obtained from the patient for publication of this case report and accompanying images, in accordance with the Declaration of Helsinki.

## Conflicts of Interest

The authors declare no conflicts of interest.

## Data Availability

The data that support the findings of this study are available on request from the corresponding author. The data are not publicly available due to privacy or ethical restrictions.

## References

[1] Limper A. H. and Prakash U. B. , Tracheobronchial Foreign Bodies in Adults, Annals of Internal Medicine. (1990) 112, no. 8, 604–609, 10.7326/0003-4819-112-8-604. 2327678

[2] Cataneo A. J. , Reibscheid S. M. , Ruiz Júnior R. L. , and Ferrari G. F. , Foreign Body in the Tracheobronchial Tree, Clinical Pediatrics. (1997) 36, no. 12, 701–705, 10.1177/000992289703601206. 9415838

[3] Kogure Y. , Oki M. , and Saka H. , Endobronchial Foreign Body Removed by Rigid Bronchoscopy After 39 Years, Interactive CardioVascular and Thoracic Surgery. (2010) 11, no. 6, 866–868, 10.1510/icvts.2010.243097, 20826558. 20826558

[4] Baharloo F. , Veyckemans F. , Francis C. , Biettlot M. P. , and Rodenstein D. O. , Tracheobronchial Foreign Bodies: Presentation and Management in Children and Adults, Chest. (1999) 115, no. 5, 1357–1362, 10.1378/chest.115.5.1357, 10334153. 10334153

[5] Hewlett J. C. , Rickman O. B. , Lentz R. J. , Prakash U. B. , and Maldonado F. , Foreign Body Aspiration in Adult Airways: Therapeutic Approach, Journal of Thoracic Disease. (2017) 9, no. 9, 3398–3409, 10.21037/jtd.2017.06.137, 29221325. 29221325 PMC5708401

[6] Thomas V. , Chandy G. , and Rolston D. D. K. , Foreign Body in the Lung for 45 Years Without Symptoms, British Journal of Diseases of the Chest. (1986) 80, no. 80, 292–294, 10.1016/0007-0971(86)90067-7. 3790422

[7] Hampton J. and StrawBlondeDesigns , Vtg 1971 Mattel Barbie Brown Plastic Accessories Set (Complete): TV, Record Player, Telephone, Drink Tray w/Cups & Suitcase-Near Mint [Internet], 2026, Etsy, https://web.archive.org/web/20260412002400/https://www.etsy.com/listing/1103794562/vtg-1971-mattel-barbie-brown-plastic.

[8] Sehgal I. S. , Dhooria S. , Ram B. , Singh N. , Aggarwal A. N. , Gupta D. , Behera D. , and Agarwal R. , Foreign Body Inhalation in the Adult Population: Experience of 25,998 Bronchoscopies and Systematic Review of the Literature, Respiratory Care. (2015) 60, no. 10, 1438–1448, 10.4187/respcare.03976, 25969517. 25969517

[9] Lin L. , Lv L. , Wang Y. , Zha X. , Tang F. , and Liu X. , The Clinical Features of Foreign Body Aspiration Into the Lower Airway in Geriatric Patients, Clinical Interventions in Aging. (2014) 9, 1613–1618, 10.2147/CIA.S70924, 25284994. 25284994 PMC4181443

[10] Jang G. , Song J. W. , Kim H. J. , Kim E. J. , Jang J. G. , and Cha S. I. , Foreign-Body Aspiration Into the Lower Airways in Adults; Multicenter Study, PLoS One. (2022) 17, no. 7, e0269493, 10.1371/journal.pone.0269493, 35793276. 35793276 PMC9258814

[11] Hamberg K. , Gender Bias in Medicine, Womens Health. (2008) 4, no. 3, 237–243, 10.2217/17455057.4.3.237, 19072473. 19072473

[12] Chapman K. R. , Tashkin D. P. , and Pye D. J. , Gender Bias in the Diagnosis of COPD, Chest. (2001) 119, no. 6, 1691–1695, 10.1378/chest.119.6.1691. 11399692

[13] Assayag D. , Morisset J. , Johannson K. A. , Wells A. U. , and Walsh S. L. F. , Patient Gender Bias on the Diagnosis of Idiopathic Pulmonary Fibrosis, Thorax. (2020) 75, no. 5, 407–412, 10.1136/thoraxjnl-2019-213968, 32054644. 32054644

